# Comparison of bilateral transversus abdominis plane block and wound infiltration with bupivacaine for postoperative analgesia after cesarean delivery

**DOI:** 10.4274/jtgga.2016.0155

**Published:** 2017-03-01

**Authors:** Ümit Görkem, Kamuran Koçyiğit, Cihan Toğrul, Tayfun Güngör

**Affiliations:** 1 Department of Obstetrics and Gynecology, Hitit University Faculty of Medicine, Çorum, Turkey; 2 Department of Anesthesiology, Hitit University Training and Research Hospital, Çorum, Turkey

**Keywords:** Cesarean delivery, transversus abdominis plane block, wound block, bupivacaine

## Abstract

**Objective:**

The study aimed to compare efficacy, safety, pain intensity and analgesic consumption in patients receiving either bilateral transversus abdominis plane (TAP) block or wound infiltration with bupivacaine after cesarean delivery (CD).

**Material and Methods:**

A total of 216 parturient women undergoing CD under general anesthesia were randomly allocated into five groups: i) controls (group 1), ii) TAP placebo (group 2), iii) TAP (group 3), iv) wound infiltration placebo (group 4), and, v) wound infiltration (group 5). Pain intensity was assessed using a visual analogue scale (VAS). Analgesic consumptions were recorded by a blinded nurse at 6, 12, and 18 hours postoperatively.

**Results:**

The baseline characteristics of the five groups were similar in terms of age, history of CD, and body mass indices (p>0.05). There were significant intergroup differences in VAS scores between all groups at the zero time-point (p=0.03), at the 6th hour (p=0.02), 12th hour (p=0.02), and at the 18th hour (p=0.02). Group 3 patients had lower pain scores and consumed less diclofenac than group 2 patients only within 12 hours postoperatively whereas pain intensity and analgesic consumption were not different between group 5 and group 4 patients. Group 5 patients received significantly less pethidine than group 4 and group 1 patients (p<0.001).

**Conclusion:**

TAP block provided better pain relief and less analgesic requirement than bupivacaine wound infiltration early after CD. Given the similar amounts of diclofenac but lower amounts of pethidine administered in the wound infiltration group, wound infiltration of bupivacaine seems promising in terms of reducing opioid use after CD under general anesthesia, especially when TAP block is not used.

## INTRODUCTION

Cesarean delivery (CD) rates have been substantially rising worldwide and Turkey is among the countries where the increasing trend is most prominent (1). The number of women showing a tendency to have CD is rising possibly because this type of delivery sounds less frightening and less painful than normal vaginal birth. CD has become an appealing option requested by women in the general population and by health care providers, owing to the concerns commonly raised about complications and pain experience during labor (2).

A painless CD is achievable using various anesthesia techniques while each technique has its own criteria to be met by the given patient. However, post-operative pain, which appears after elimination of the anesthesia, continues for days after surgery and still constitutes a major problem in patients receiving CD (3). It was reported that about 10% of women still experience substantial pain after CD even though a programmed analgesic regimen was implemented (4). Women with depressive symptoms during the postnatal period report pain more commonly than those without depression (5). Furthermore, high pain levels after CD was reported to be associated with loss of ability for breast feeding and taking care of the newborn (6).

Transversus abdominis plane (TAP) block has gained popularity among physicians owing to the ease of the procedure and its effectiveness in reducing pain after lower abdominal surgery. In recent years, there have been a number of comparative studies demonstrating its effectiveness in postoperative pain control after CD under spinal anesthesia (7, 8). Wound infiltration of non-steroid anti-inflammatory drugs or local anesthetics during wound closure is an alternative for providing pain relief after CD. A systematic review of studies on wound infiltration of local anesthetics during CD under spinal anesthesia established that the technique provided a significant decrease in morphine consumption (9). Data is limited regarding its effectiveness in patients receiving general anesthesia. Even a single injection of local anesthetics within wound layers was shown to decrease morphine requirements within 12 hours after CD (10). Therefore, we conducted a placebo-controlled study to compare the efficacy and safety of these two aforementioned techniques in patients undergoing primary elective CD under general anesthesia.

## MATERIAL AND METHODS

The Ethics Committee of Ankara Numune Hospital approved the study, which was conducted in accordance with the Declaration of Helsinki 2013 Brasil version (March 27. 2014, 20796219-E-14-159). All patients gave written informed consent to take part in the research. This prospective randomized double blinded five-arm study was conducted at the Hitit University Hospital and comprised patients undergoing elective CD between April and August 2014. Eligible patients were those at minimum 37 weeks of gestational age, with American Society of Anesthesiologists physical status I-II, aged between 18-45 years, non-laboring at the time of allocation, and those requesting general anesthesia. Patients with a body mass index (BMI) of more than 40 kg/m^2^, with a history of chronic pain, drug abuse, cardiac and pulmonary disease, and those undergoing emergency CD were excluded. Based on the above criteria, a total of 216 patients were randomly allocated into two treatment and two placebo groups and one control group, according to randomly sequenced numbers generated using a computer-based random number generator in blocks of two methods, to ensure a near-equal distribution of patients into treatment arms. Group allocation was concealed with sealed envelopes including the code of the group that an individual patient would be included in. The patient and the investigator who collected study data were blinded to the group allocation.

The patients were divided into five groups so as to receive the planned procedure for them: i) group 1 (G1)- controls, ii) group 2 (G2)- TAP placebo, iii) group 3 (G3)- TAP, iv) group 4 (G4)- wound infiltration placebo, and, v) group 5 (G5)- wound infiltration. G1 served as controls and received no additional procedures. G3 patients received ultrasound (US)-guided TAP block with 20 mL of 0.25% bupivacaine after closure of the wound. Likewise, G5 patients received wound infiltration of 20 mL of 0.25% bupivacaine before closure of the wound. G2 patients received a 20 mL physiologic saline injection identical to G3, and G4 patients received a 20 mL infiltration of physiologic saline identical to G5.

In the operating room, standardized monitoring including electrocardiogram, non-invasive blood pressure, and pulse oximetry was provided before induction of anesthesia. Anesthesia was induced with 5 mg/kg of thiopental sodium and orotracheal intubation was performed. In all patients, skin preparation and sterile draping included whole upper and lower abdomen to allow for bilateral anterolateral wall approach of the abdomen. Anesthesia was maintained using sevoflurane with an end-tidal concentration of 2.5%.

CD procedures were performed by the same surgeon who used the same CD technique. After delivery, the surgeon operating on the patient continued in sterile conditions in all groups and performed all procedures himself after ensuring the investigator who would be involved in data collection was outside the operating room. In the TAP block group (G3), after closing the wound, the anesthesiologist performed the TAP block as described previously by Costello et al. (11). A linear transducer US probe was placed midway between the costal margin and the iliac crest at the anterolateral wall of the abdomen and fasciae of the external oblique, internal oblique and transversus abdominis muscles and the TAP was identified. A 22-gauge 80-mm aspiration needle was introduced into the TAP under real-time US guidance. After confirmation of the needle position by injecting 1 mL of test dose and negative aspiration, 20 mL of 0.25% bupivacaine was injected with negative aspirations performing at every 5 mL. The procedure was repeated on the contralateral side. In the TAP placebo group (G2), the procedure was identical to that in G3 except for injecting 20 mL of normal physiologic saline after entering the TAP. The skin was covered at needle insertion sites with dressing to ensure blinding.

In the wound infiltration group (G5), infiltration was performed before closure of the wound. The surgeon administered 20 mL of 0.25% bupivacaine within the fascia and also to the subcutaneous fat tissue above the fascia, as described previously by Niklasson et al. (10). In the wound infiltration placebo group (G4), the procedure was identical to that in G5 except for infiltrating 20 mL of normal physiologic saline to the fascia. In the control group (G5), no additional procedures were performed after delivery and the surgeon proceeded to skin closure without any delay. In G1, G4, and G5 where TAP procedure was not performed, sham skin dressings were applied at sites corresponding to needle insertion sites for TAP to ensure blinding of the data collectors.

After completion of the treatment, 0.1 mg/kg of morphine and 15 µg of sufentanil was given. All patients received 2.5 mg of neostigmine to eliminate any residual neuromuscular blockade. The patients were extubated soon after they opened their eyes on command and spontaneously breathing. In the postoperative care unit, patients received no type of patient-controlled anesthesia (PCA). Patients received a multimodal analgesic regimen including intramuscular administration of diclofenac sodium (Dikloron 75 mg, Deva Drugs, Turkey) and pethidine (Aldolan Gerot 100 mg, Liba Drugs, Turkey). Nausea and vomiting was treated using intramuscular metoclopramide 10 mg, as needed. Patients were asked by a blinded investigator to rate their pain using a 100-mm visual analogue scale (VAS) (0 hours) before receiving the first administration dose of 75 mg intramuscular diclofenac on arrival in the postoperative care unit. Patients were strongly advised that should ask for additional analgesics if needed at any time after surgery. For pain not relieved with diclofenac, pethidine 50 mg muscular injection was administered. Repeated doses of diclofenac and pethidine were administered if pain was not relieved, avoiding excessive doses of the same analgesics. Analgesic use was recorded by a blinded nurse at 6, 12, and 18 hours postoperatively, and the pain assessment was repeated to record the worst VAS score by requesting the patient to cough or change their position from supine to sitting.

### Statistical analysis

A priori sample size calculation was performed for a five-group fixed effects one-way ANOVA test. The effect size (Cohen’s f) in this study was found as 1.75 (12). According to Cohen (13) an effect size of more than 0.40 was defined as large. Therefore, the effect size in that study was considered as large and was not used. In G power, assuming a medium effect size (f=0.25), with a significance level of α=0.05, and β=0.20, we found that 40 subjects were required in each group.

Statistical analyses were performed using the Statistical Package for the Social Sciences version 21 (SPSS IBM Inc. Chicago, USA). The distribution of variables was tested using visual histograms and the Kolmogorov-Smirnov test to determine normality. Descriptive statistics for continuous variables were reported as mean ± standard deviation and categorical variables were represented as frequency and percentage. Categorical variables were compared using Chi-square test or Fisher's exact test where appropriate. One-way ANOVA or Welch ANOVA were used to compare normally distributed continuous variables among the five groups, based on the homogeneity of variances, which was tested using the Levene test. Post-hoc tests were performed using Hochberg’s or Tamhane test according to the presence of homogeneity. Dunn-Sidak’s method was used to calculate the level of significance for multiple five-group comparisons [p<1-(1-0.05)1/5=0.0102 was considered as statistically significant].

## RESULTS

The baseline characteristics of the patients are given in Table 1. The five groups were similar in terms of age, history of CD and BMIs. The procedure for TAP block took a mean of 4.6±0.8 min and 4.2±0.9 min in patients who received local analgesic and placebo, respectively (p=0.74). The procedure for wound infiltration of bupivacaine took 2.8±0.4 min and 2.9±0.6 min in patients receiving local analgesic and placebo, respectively (p=0.86). No patients had complications during application of TAP injection under US guidance. TAP injections caused no hematoma, bleeding or pain sensitivity at the site of application.

There were significant intergroup differences in VAS scores between the treatment, placebo, and control groups at the zero time point (p=0.03), at 6 hours (p=0.02), 12 hours (p=0.02), and at 18 hours (p=0.02) as shown in Table 2. At the zero time point, patients in G3 reported significantly lower VAS scores than those in G5 (p=0.021), G2 (p=0.039), G4 (p<0.001), and the control group (G1) (p=0.009). The Post-hoc Tamhane test indicated that the difference between TAP block and wound infiltration placebo groups were the most pronounced. At the 6^th^ hour, patients in TAP block group reported significantly lower VAS scores than those in the TAP placebo group (p=0.008), wound infiltration placebo group (p=0.004), and control group (p=0.02). When the Hochberg post-hoc test was applied, the most pronounced difference was found between the TAP block and wound infiltration placebo groups. At the 12^th^ hour, patients in the TAP block group reported significantly lower VAS scores than those in TAP placebo group (p=0.017), and patients in the wound infiltration group reported significantly lower VAS scores than those in the TAP placebo group (p=0.017). As the Hochberg post-hoc test revealed, the difference between TAP block and TAP placebo groups were the most pronounced. At the 18^th^ hour, patients in the TAP block group reported significantly lower VAS scores than those in the wound infiltration placebo group (p=0.02) and in the control group (p=0.002). Again, the Hochberg post-hoc test designated that the most pronounced difference was between TAP block and the control groups.

The analgesic requirements after surgery are summarized in Table 3. There were significant intergroup differences in diclofenac (p=0.004) and pethidine use (p<0.001) of the patients. According to the post-hoc pairwise comparisons for diclofenac use, patients in TAP block group used significantly less diclofenac than those in the wound infiltration group (p=0.007), TAP placebo group (p<0.001), and wound infiltration placebo group (p=0.002) where the difference between TAP block and TAP placebo groups were the most pronounced one. According to the post-hoc pairwise comparisons for pethidine use, patients in the TAP block group required significantly less pethidine than those in the TAP placebo group (p<0.001), wound infiltration placebo group (p<0.001), and control group (p<0.001). Also, patients in the wound infiltration group used significantly less pethidine than those in the TAP block placebo group (p=0.002), wound infiltration placebo group (p=0.009), and control group (p=0.004) in which the difference between TAP block and TAP placebo groups was again the most pronounced.

## DISCUSSION

Our study showed that a single injection TAP block satisfactorily provided pain relief for 12 hours postoperatively in patients who underwent elective CD under general anesthesia whereas such benefit was limited in patients who received wound infiltration with local anesthetic at similar doses. TAP block provided the lowest VAS scores in all assessments with the largest difference being observed immediately after the patients arrived in the postoperative intensive care unit. The difference in VAS scores observed between TAP block and TAP placebo patients sustained until the 12^th^ hour assessment although patients in the TAP placebo group received much more diclofenac and pethidine. The difference between the TAP block and TAP placebo groups was not sustained and receded at the 18^th^ hour, indicating the rapid onset but short duration effect of bupivacaine when used in TAP block.

There have been several studies on TAP block in patients undergoing CD under spinal anesthesia (14). Although some of these showed no benefit and implied that TAP block may have a potential role in pain relief following general anesthesia, few studies have focused on this topic (11, 15). Continuous wound infiltration of local anesthetics has also gained popularity in recent years; however, it is yet to be established whether a single-dose wound infiltration of local anesthetics provides benefit in terms of postoperative pain relief.

In a study by Eslamian et al. (16) VAS scores were significantly lower over time (within 24 h) in patients who received TAP block after CD with general anesthesia. These authors also reported that patients in the TAP block group requested analgesics after a longer time than those in the control group. Similar to us, these investigators did not use PCA in their patients. In another study on patients who underwent CD under general anesthesia, Tan et al. (17) reported similar outcomes; they observed a significant decrease in morphine use in the TAP block group, whereas VAS scores were not different between patients in the TAP block and standard care groups. In their study, principal component analysis was implemented as a routine standard of practice, whereas such protocol is not in use in our institution. Based on the above facts, we postulate that TAP block is beneficial both in terms of limiting analgesic use and improving pain relief in patients undergoing CD under general anesthesia.

In our study, we found no differences between patients receiving wound infiltration of 20 mL of bupivacaine or placebo in regards to VAS scores. Although we observed a slight difference at the 6^th^ and 12^th^ hours postoperatively, the difference did not reach statistical significance. In a randomized controlled trial, Niklasson et al. (10) injected a single dose 40 mL (2.5 mg/mL) of bupivacaine and adrenaline into the fascial layers before closure of the wound after CD and compared these patients with placebo controls. In that study, wound infiltration with bupivacaine resulted in lower morphine requirement for the first 12 postoperative hours and also a lower pain intensity for 6 hours. Supporting this, in our study, diclofenac use did not differ between the bupivacaine and placebo groups, whereas patients in the bupivacaine group used significantly lesser amount of pethidine in the postoperative period.

In the present study, patients in the TAP block group had lower VAS scores than those in the wound infiltration group soon after the operation. However, the significance of the difference was not sustained at the 6^th^ and 18^th^-hour assessments. Moreover, the difference in the mean amount of pethidine use was not significantly different between these two groups, whereas patients in the TAP block group used significantly less diclofenac than those in the wound infiltration group. Given the similar baseline characteristics of the patients in the two groups and similar amount and dose of bupivacaine used for both protocols, we postulate that TAP block provides better pain relief than wound infiltration soon after the operation only, but its benefit in terms of reducing opioid use should be questioned in further research. The reason why TAP block provided lower postoperative pain than wound infiltration may be explained by its better pain control effect. With TAP block, the anesthetic directly blocks the afferent nerves before these nerves enter the anterior abdominal wall. Visceral pain relief may be due to posteromedial diffusion of the anesthetic along the fascial plane. To our knowledge, no other studies have compared TAP block with wound infiltration of local anesthetics for postoperative pain relief after CD.

One may raise concern about the high number of elective cesarean deliveries under general anesthesia in this study. This is due to the low number of anesthesiologists in our hospital and reluctance of the pregnant women to regional anesthesia for CD. In one review on TAP block for transverse lower abdominal incisions, 8 out of 12 trials were on patients receiving CD (18). Among these, only two studies included patients who underwent surgery under general anesthesia. Similarly, in one review on patients undergoing wound infiltration during CD, only one out of 12 studies was on patients receiving general anesthesia (9). This inconsistency may be due to fact that women in our country are likely to have a tendency towards undergoing a totally painless and unconsciousness experience during birth, which makes postoperative pain control even more important.

A study from Japan by Tsuchiya et al. (19) -reported that combining TAP block with general anesthesia promoted intraoperative hemodynamic stability in patients with severe cardiovascular disease. Although this was not valid for our study population, TAP block seems to provide an advantage for patients with severe hemodynamic instability.

In parallel with our study, Tharwat et al. (20) assessed the efficacy and safety of incisional infiltration of local lidocaine in patients undergoing CD. They demonstrated that lidocaine administration reduced the opioid analgesic dose postoperatively and enhanced patient recovery.

The main strength of the study was that, to our knowledge, there have been no comparative studies performed to investigate whether TAP block or wound infiltration of local anesthetics provides better postoperative analgesia after CD. There were certain limitations in our study. First, because patients in the TAP block and TAP placebo group had pain sensitivity at the needle insertion site and might have reported pain during the investigator assessment, a flawless blinding might not have been achieved in this study. Also, despite being strongly advised, patients might not always have asked for analgesics from nurses because of individual variations in pain conception and resistance against pain. Another limitation of the present study was that analgesic use was not quantified as doses per weight and per time, which may produce more accurate implications from study outcomes. The comparison of equal doses of bupivacaine given through two distinct routes of administration is also questionable because different routes of administration would have produced different amounts of drug distribution through the abdominal wall.

In conclusion, TAP block provided better pain relief and less analgesic requirement than bupivacaine wound infiltration early after CD under general anesthesia. Given the similar amounts of diclofenac but lower amounts of pethidine used in the wound infiltration group, wound infiltration of bupivacaine seems promising in terms of reducing opioid consumption after CD under general anesthesia, especially when TAP block is not used.

## Figures and Tables

**Table 1:**
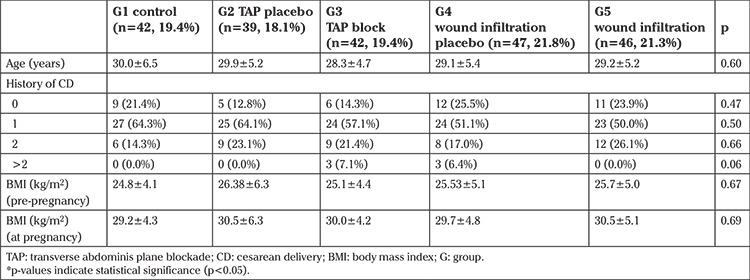
Baseline characteristics of the patients

**Table 2:**
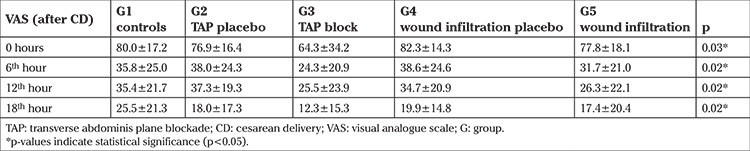
Visual analogue scale, scores of the patients

**Table 3:**
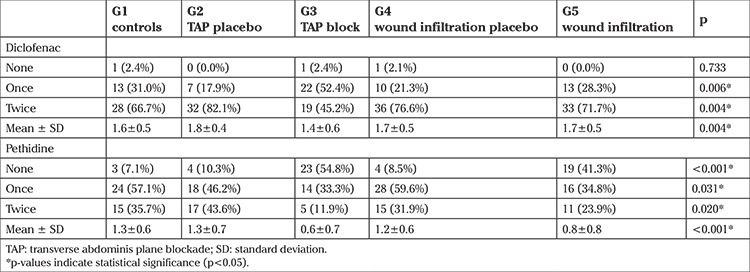
Analgesic requirements after surgery
